# Specific location of galactosylation in an afucosylated antiviral monoclonal antibody affects its FcγRIIIA binding affinity

**DOI:** 10.3389/fimmu.2022.972168

**Published:** 2022-10-12

**Authors:** Grayson Hatfield, Lioudmila Tepliakova, Genevieve Gingras, Andrew Stalker, Xuguang Li, Yves Aubin, Roger Y. Tam

**Affiliations:** Centre for Oncology, Radiopharmaceuticals and Research, Biologics and Radiopharmaceutical Drugs Directorate, Health Canada, Ottawa, ON, Canada

**Keywords:** monoclonal antibody, glycosylation, effector activity, 2D NMR, structure-function activity

## Abstract

Monoclonal antibodies (mAbs) comprise an essential type of biologic therapeutics and are used to treat diseases because of their anti-cancer and anti-inflammatory properties, and their ability to protect against respiratory infections. Its production involves post-translational glycosylation, a biosynthetic process that conjugates glycans to proteins, which plays crucial roles in mAb bioactivities including effector functions and pharmacokinetics. These glycans are heterogeneous and have diverse chemical structures whose composition is sensitive to manufacturing conditions, rendering the understanding of how specific glycan structures affect mAb bioactivity challenging. There is a need to delineate the effects of specific glycans on mAb bioactivity to determine whether changes in certain glycosylation profiles (that can occur during manufacturing) will significantly affect product quality. Using enzymatic transglycosylation with chemically-defined *N*-glycans, we show that galactosylation at a specific location of *N*-glycans in an afucosylated anti-viral mAb is responsible for FcγRIIIA binding and antibody-dependent cell-mediated cytotoxicity (ADCC) activity. We report a facile method to obtain purified asymmetric mono-galactosylated biantennary complex *N*-glycans, and their influence on bioactivity upon incorporation into an afucosylated mAb. Using ELISA, surface plasmon resonance and flow cytometry, we show that galactosylation of the α6 antenna, but not the α3 antenna, consistently increases FcγRIIIA binding affinity. We confirm its relevance in an anti-viral model of respiratory syncytial virus (RSV) using an adapted ADCC reporter assay. We further correlate this structure-function relationship to the interaction of the galactose residue of the α6 antenna with the protein backbone using 2D-^1^H-^15^N-NMR, which showed that galactosylation of at this location exhibited chemical shift perturbations compared to glycoforms lacking this galactose residue. Our results highlight the importance of identifying and quantifying specific glycan isomers to ensure adequate quality control in batch-to-batch and biosimilar comparisons.

## Introduction

Monoclonal antibodies (mAbs) are an important class of glycoprotein-based biotherapeutics that are used to treat cancer, inflammation and viral disease, of which many marketed products are of the IgG1 class (1). IgG1 glycoproteins comprise heavy and light chains that form the variable (Fab) and constant (Fc) regions with an approximate molecular weight of 150 kDa, with each region having distinct biological activities (2). The Fab regions are typically designed to bind to a target molecule (some of which are receptors) to inhibit its function, while the Fc region can initiate downstream immunological responses by various immune cells to further affect the biological activity of mAbs such as antibody-dependent cellular cytotoxicity (ADCC). The Fc region is structurally similar amongst IgG1 molecules and has a single *N*-glycosylation site at N297 of each of the two heavy chains. Protein glycosylation is a post-translational modification that is dynamically regulated by numerous glycosyltransferase and glycosidase enzymes during protein synthesis in the endoplasmic reticulum and Golgi bodies, and by these enzymes that may be in the cell or in the extracellular matrix (3). As such, manufacturing and production of mAbs typically result in heterogeneous mixtures of glycan structures, whose composition is sensitive to numerous expression parameters such as pH, temperature and media composition (4).

Specific *N*-glycan structures can greatly influence mAb bioactivity by altering its binding affinity to Fc receptors (FcR) on the surface of immune cells. Amongst the most widely studied glycan structure is the core fucose residue in *N*-glycans, where its presence decreases ADCC activity (5, 6) by hindering binding to the FcγRIIIa (CD16A) receptor (7, 8). Galactose is another important terminal carbohydrate residue that is directly proportional to ADCC, with previous studies showing inconsistent results (9–16). Although these insightful studies highlight the overall effect of fucosylation and general galactosylation on the interaction between Fc and FcγRIIIa, the use of non-homogeneous or ambiguous galactosylated glycoforms limits its exact correlation to its biological activity. Recent advances in glycoengineering using enzymatic transglycosylation have enabled the synthesis of mAbs with chemically-defined glycans and perform more detailed structure function analyses (12, 17–21). A recent study has reported that in glycoengineered rituximab (a commercially-available anti-inflammatory mAb) comprising defined homogeneous glycoforms, the location of the galactose residue in fucosylated biantennary complex Fc *N*-glycans showed differing activity (21); a galactose residue on the α6-antenna showed greater FcR binding and ADCC activity compared to that in the absence of the α3 antenna. This difference in activity is important in the regulatory evaluation of mAb therapeutics, as different commercially-available mAbs have been shown to have varying degrees of each mono-galactosylated isomers (22). However, whether this difference in activity between mono-galactosylated isomers is also observed in non-fucosylated glycans and in other mAbs targeting other antigens has not been reported. Further, it is unclear how these changes using chemically-defined glycans affect the Fc protein structure at the molecular level. Understanding how these epitopes affect mAb bioactivity and correlation to their structural properties is important, as glycoengineering is emerging as a strategy in mAb drug design and glycan characterization is crucial to ensure mAb efficacy and safety (23, 24).

While several glycosylation structure-function studies have been performed on mAbs in the context of anti-cancer and anti-inflammatory diseases (12, 17–21), those that focus on anti-viral mAbs have remained limited (10, 25). With the increasing development of anti-viral mAbs, especially with mAbs targeting SARS-CoV2 (26), it is important to continue to increase the knowledge about how glycosylation affects mAbs targeting anti-viral diseases. To this end, we report how mAb glycosylation affects its activity towards Respiratory Syncytial Virus (RSV), a virus that causes respiratory illness primarily in children, the elderly, and immunocompromised individuals, and is often misdiagnosed as influenza (27). RSV infects bronchial epithelial cells and causes increased mucous production that restricts breathing. While RSV vaccines were first produced for over 30 years, they are ineffective and have even been shown to exacerbate the disease (28). Viral Infections occur *via* binding of RSV protein F (RSV-F) and RSV-G to host cell surfaces (29, 30) that permits viral entry into the cells. A monoclonal antibody (mAb) therapeutic, palivizumab (Synagis), is an RSV-F neutralizing mAb that is effective prophylactic treatment, especially in vulnerable patients such as premature and young infants (31). Palivizumab functions by inhibiting the binding of RSV-F to its host cell surface receptor and therefore inhibits viral entry into the cell. Hiatt et al. previously reported that palivizumab produced in plant cells (*Nicotiana benthamiana*) yields afucosylated agalactosylated complex (G0, 76%) and high mannose glycans (Man7/9, 17%), which increased FcγRIIIa binding affinity and decreased viral titre in the lungs of Cotton Rats (25). However, a more detailed study on how glycosylation affects palivizumab has not been reported. Herein, we report that FcγRIIIa binding and ADCC activity is significantly increased by galactosylation of the α6 antenna (but not the α3 antenna) of afucosylated biantennary glycans in palivizumab. Using enzymatic transglycosylation to engineer afucosylated mAbs with chemically-defined glycans with varying degrees of galactosylation at specific locations, the changes in binding affinity to FcγRIIIa receptors were assessed using ELISA, surface plasmon resonance (SPR), flow cytometry and an ADCC assay. In order to support glycan-associated bioactivities measurements, we have characterized each glycoform using 2D-^1^H-^15^N-NMR fingerprints on isotopically labelled Fc domains to assess the location of the terminal galactose moieties.

## Results

### Synthesis of afucosylated palivizumab with defined glycoforms

The glycoprofile of commercially available palivizumab (Synagis) was first characterized using high pH anionic exchange chromatography-pulsed amperometric detection (HPAEC-PAD). Palivizumab was treated with the endoglycosidase PNGaseF to release free *N*-glycans that were measured directly *via* HPAEC-PAD. In our analysis, commercially-available palivizumab comprises predominantly G0F, G1F and G2F (25, 46 and 17% respectively) by HPAEC-PAD analysis (**Supplementary Figures S1A, B**). Surprisingly, HPAEC-PAD analysis revealed 4% of a high mannose (Man-5) glycan and pseudohybrid *N*-glycan structures, which were not detected by intact mass spectrometry analysis, nor reported previously. Intact mass spectrometry shows the presence of both symmetric and asymmetric glycosylation of the dimerized Fc heavy chains. Based on previous literature results (25) and our HPAEC-PAD analysis of PNGase F-released glycans (**Supplementary Figures S1A, B**), the peak at 147,958.813 Da corresponds to both glycans being of the agalactosylated G0F structure, while peaks at 148,118.953 and 148,440.328 Da indicate the asymmetric glycosylation comprising agalactosylated/monogalactosylated (G0F/G1F) and mono-/digalactosylated (G1F/G2F) structures, respectively (**Supplementary Figures 1C, D**).

**Figure 1:**
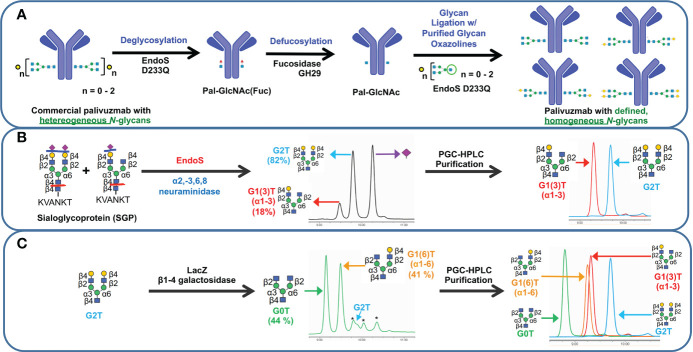
Schematic representation of transglycosylation of palivizumab to generate homogeneous glycoforms. **(A)** Deglycosylation of commercially-available palivizumab using EndoS D233Q generates the truncated GlcNAcFuc disaccharide (blue square = GlcNAc; red triangle = fucose) remaining on palivizumab. Defucosylation is achieved using fucosidase GH29, and subsequent glycan ligation of purified glycan oxazoline is achieved using EndoS 233Q. **(B)** Enzymatic reaction and purification of truncated glycans (mono-galactosylated at the α3 antenna (G1 (3)T) and digalactosylated (G2T)) using EndoS and α2-3,6,8 neuraminidase to cleave truncated glycans between the reducing end chitobiose core, followed by purification by porous graphitic carbon (PGC)-HPLC. **(C)** Synthesis of truncated agalactosylated (G0T) and monogalactosylated at the α6 antenna (G1 (6)T) glycans using LacZ β1-4 galactosidase (* indicates reaction byproduct peaks), followed by purification by PGC-HPLC. HPAEC-PAD trace of overlaid spectra of purified glycans are shown on the right in each of **(B, C)**.

With our goal to perform glycosylation-based structure-function studies on palivizumab, we used a glycoengineering strategy involving enzymatic transglycosylation (17, 32). Although several EndoS endoglycosidases have been reported with slightly varying substrate specificities (17, 19), we chose EndoS D233Q mutant, which does not affect mannose (Man5) glycans, and used this as an internal standard to further measure completeness of the transglycosylation reaction. Cleavage of the chitobiose core of complex *N*-glycans (*i.e.* between the two GlcNAc residues closest to the N297 position of the protein backbone) with EndoS D233Q leaves the palivizumab protein backbone with a single GlcNAc and a core-1,6-fucose residue, on each heavy chain (Pal-GlcNAc(Fuc), **Figure 1A**), as well as the aforementioned 4% uncleaved Man 5 glycans. Subsequent treatment with fucosidase GH29 cleaved the core fucose residue to afford the afucosylated analogue (Pal-GlcNAc) that could then undergo transglycosylation with purified glycan oxazoline compounds and EndoS D233Q. Of note, the three steps were performed sequentially in a one-pot reaction without the need for purification until the completion of the transglycosylation reaction steps.

To obtain biantennary glycan analogues with varying degrees of galactosylation, sialoglycoprotein (SGP) was first extracted from egg yolk (33) and treated with EndoS and α2-3,6,8 neuraminidase to cleave the glycans between the chitobiose core, and removal of the terminal sialic acid residues, respectively, **Figure 1B**. In addition to the production of the truncated digalactosylated (G2T) glycan, we observed the formation of a monogalactosylated glycan (18%) that was also previously reported, but the specific location of its terminal galactose residue was not identified (34). To this end, we purified the glycans by preparative porous graphitic carbon (PGC)-HPLC, a powerful technique that is able to separate glycan isomers as well as their respective anomers (35, 36), and confirmed glycan purity and structural identity by HPAEC-PAD and tandem mass spectrometry (**Figure 1B**, right panel, and **Supplementary Figure S2A**). A recent report showed that branch-specific glycan residue characterization can be achieved using positive mode CID MS/MS fragmentation, where the relative peak intensities of the MS/MS-fragmented glycan peaks are compared between two separate purified glycan biantennary branching isomers; MS/MS-induced cleavage of the α3 antenna between the GlcNAc-β1-2-Man linkage produces a relatively higher abundance of the cleaved and its complementary glycan fragments compared to cleavage of the α6 antenna (37). It is noteworthy that this fragmentation will occur at both α3 and α6 antennae, albeit at different rates, resulting in the presence of both cleaved peaks in the MS/MS spectra of a single compound; therefore MS/MS analysis of a single isomeric compound is insufficient for assigning antenna-specific glycan residues, and purified compounds of both isomers are required to enable antenna assignment. Thus, we required the synthesis of a glycan comprising monogalactosylation at a known antenna. It has been well characterized that LacZ β1-4 galactosidase can selectively cleave the Gal-β1-4-GlcNAc residue of the α3 antenna to yield monogalactosylation at the α6 antenna (38, 39), and thus we treated our purified G2T with LacZ β1-4 galactosidase, **Figure 1C**. Further extended reaction with this enzyme resulted in the formation of the truncated agalactosylated G0T glycan. PGC-HPLC purification of each glycan analogue following the LacZ reaction eluted the anomers of each glycan at different retention times (See Materials and Methods), which were also different than the previously unidentified monogalactosylated truncated glycan from the SGP isolation, indicating that the latter glycan was not monogalactosylated at the α6 antenna. The β- and α- anomers of SGP-derived monogalactosylated glycan eluted at approximately 23.0 and 28.0 min, while the anomers of G1 (6)T eluted at approximately 26.0 min and 31.5 min. As a further comparison, **Figure 1C** (right panel) shows the overlaid HPAEC-PAD chromatograms of the four PGC-HPLC-purified glycans with varying degrees of galactosylation; importantly, the two monogalactosylated glycans isolated using either the SGP isolation or LacZ β1-4 galactosidase treatment possessed different retention times. With the two purified monogalactosylated glycans in hand, comparative tandem MS/MS analysis revealed that the monogalactosylated product isolated from SGP had a higher relative abundance of the cleaved GalGlcNAc ([GalGlcNAc+H]^+^, m/z 366.07) and its complementary fragment ([M-GalGlcNAc+H]^+^, m/z 911.17, **Supplementary Figure S2A**, pink peaks) compared to the cleavage of the terminal GlcNAc of the opposite antenna ([GlcNAc+H]^+^, m/z 204.02) and its complementary fragment ([M-GlcNAc+H]^+^, m/z 1073.23, **Supplementary Figure S2A**, yellow peaks); conversely, an opposite relative abundance of these peaks are observed with the known α6-monogalactosylated product obtained following LacZ β1-4 galactosidase cleavage (**Supplementary Figure S2B**). Therefore, the previously unidentified monogalactosylated glycan isolated from SGP is assigned to be that of the α3 antenna.

Purified glycans were reacted with 2-chloro-1,3-dimethylimidazolinium chloride (DMC) and trimethylamine to obtain the glycan oxazoline precursors that were then used with EndoS D233Q for transglycosylation. Glycan-remodelled palivizumab were purified using Protein A resin, which included washing with 0.5 M sucrose solutions prior to mAb elution to remove unreacted glycan starting materials.

Completion of the reactions were monitored by SDS-PAGE and intact mass spectrometry, **Supplementary Figure S3**. Cleavage by PNGaseF further shows that the afucosylated glycans present in the remodelled palivizumab analogues are homogeneous and that the original chitobiose linkage to the asparagine residue (GlcNAc β1-4-GlcNAc-N297) was formed properly, **Supplementary Figure S4**. Importantly, the percentage of Man5 residues were between 2-3% compared to the ligated glycan for all four analogues, indicating that the degree of transglycosylation is between 97-98% for each glycoform.

### Binding assays and biological activity to FcγRIIIA

**Figure 2:**
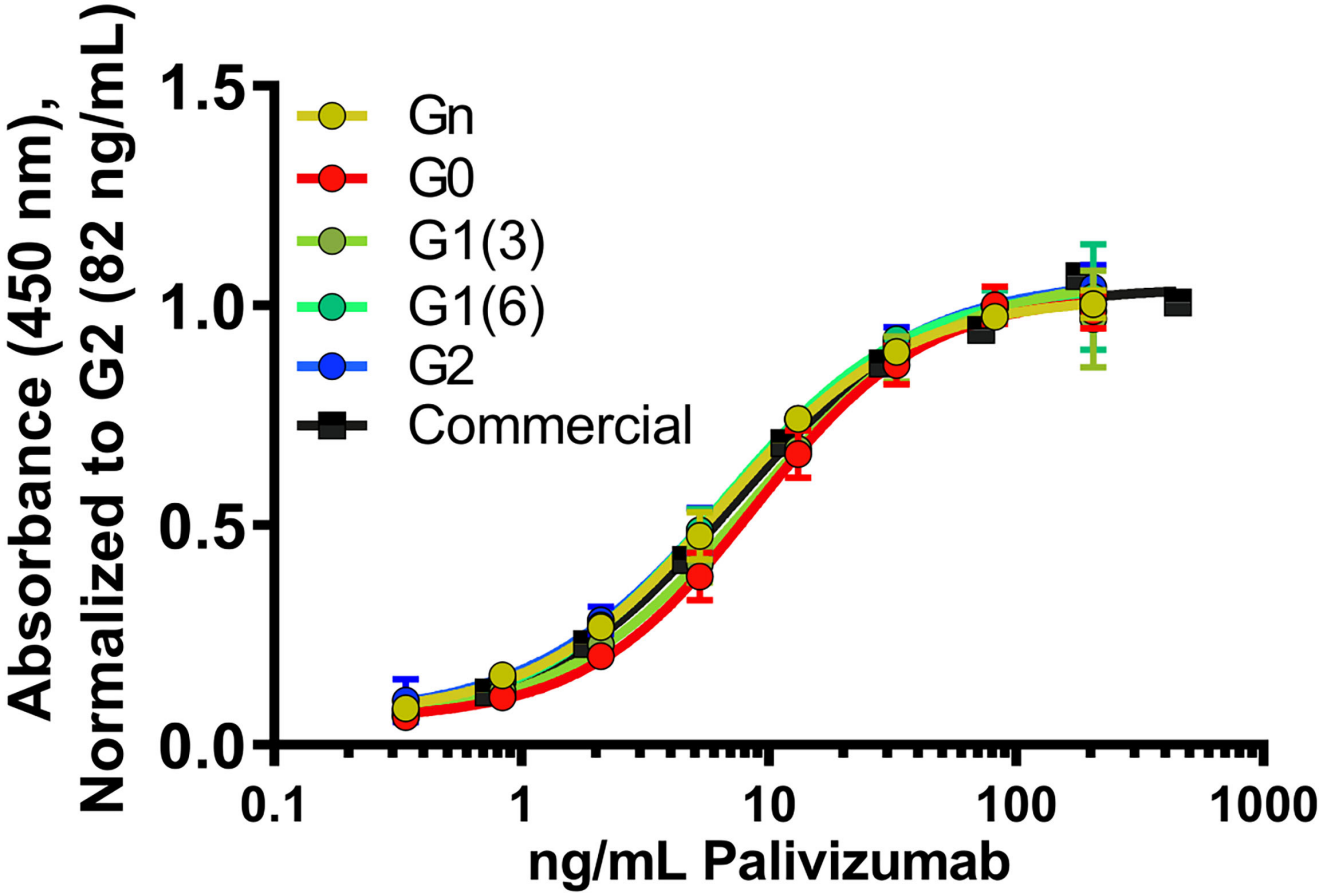
Fab binding of glycan-remodeled palivizumab to RSV-A2 F-protein is not affected by remodelled Fc-glycans. Comparable dose-response curves are obtained in the various glycoform analogues in an ELISA assay. RSV-A2 F-protein was coated at 0.25 μg/mL, followed by mAb binding. An anti-human Fc-HRP and Ultra-TMB were used for detection at 450 nm. Error bars show mean ± standard deviation (n=3 replicates); non-linear regression (4-PL) was used for curve-fitting in GraphPad. EC50 values generated and statistically analyzed (One-way Anova, Tukey *post-hoc* analysis) by GraphPad showed no significant differences between the various groups (p = 0.3184).

To assess the role of varying glycan structures on palivizumab, we first examined whether changes in Fc glycosylation affects the binding of the Fab region to its targeted RSV F-protein, using RSV-F recombinant protein-immobilized ELISA. Consistent with previous reports, varying galactosylation or fucosylation levels by enzymatic transglycosylation did not affect Fab binding, as EC50 values were not statistically significant between commercial (ie. most fucosylated), deglycosylated and afucosylated glycans with varying galactosylation (p=0.3184, **Figure 2**).

**Figure 3:**
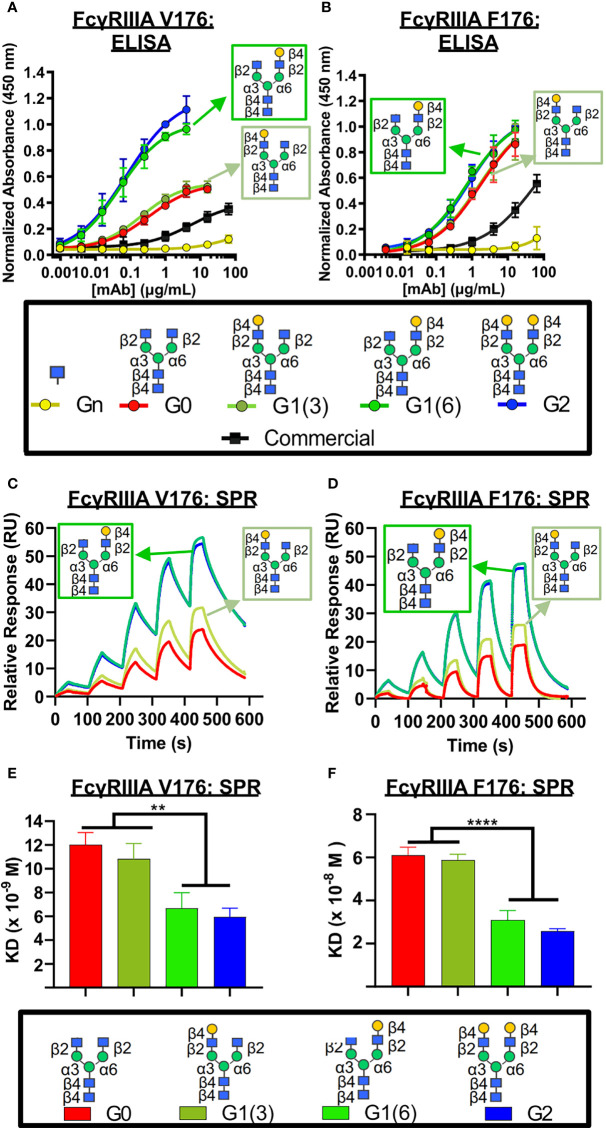
FcγRIIIa (CD16A) binding shows galactose on the α6 antenna significantly affects activity – 2D binding assays. **(A,B)** Dose response curves are obtained in the various glycoform analogues in an ELISA assay. **(A)** CD16A V 176 (high affinity) or **(B)** CD16A F176 (low affinity) were coated, followed by mAb binding. An anti-human Fab-HRP antibody, and Ultra-TMB were used for detection at 450 nm. Error bars show mean ± standard deviation (n=4 replicates); non-linear regression (variable slope, four parameters) was used for curve-fitting in GraphPad. **(C-F)** Surface Plasmon Resonance (SPR) of each palivizumab glycoform for either **(C, E)** CD16A V176 or **(D, F)** CD16A F176. **(C, D)** Overlaid sensograms and **(E,F)** dissociation constant (KD, M) are shown. For (**E, F**), error bars show mean KD ± standard deviation from 3 replicate binding experiments; **p < 0.01; ****p <0.0001, one-way ANOVA, Tukey *post-hoc* analysis.

Next, we examined the influence of the terminal galactose residues of afucosylated Fc glycans on binding to the higher affinity isoform (V176) of FcγRIIIa (CD16A), a receptor primarily involved in ADCC. Using ELISA (**Figure 3A**), a significantly higher binding of all non-fucosylated glycans was observed compared to the commercially available palivizumab that comprises mostly fucosylated G0F and G1F glycans. Intriguingly, comparison amongst only afucosylated glycoforms revealed a higher binding activity in afucosylated glycans bearing a terminal galactose residue at the α6 antenna (i.e. G1 (6) and G2), compared to those that lack a galactose residue at this position (i.e. G0, G1 (3), **Figure 3A**). EC50 values of G0 and G1 (3) glycoforms were not determined, as they never reached maximum saturation compared to G1 (6) and G2 glycoforms. The presence of a single GlcNAc residue following EndoS and fucosidase treatment (i.e. Gn glycoform) exhibited minimal binding. We observe a similar trend for binding to the lower-affinity FcγRIIIA F176 variant, although the differences between the various galactosylation states are less pronounced compared to the high-affinity V176 isoform; statistical analysis of EC50 values reveals that the absence of a galactose residue on the α6 antenna was generally statistically different (p < 0.0404, with the exception of monogalactosylated G1 (3) vs G1 (6), p = 0.059, **Figure 3B**).

We further confirm these observations using surface plasmon resonance (SPR) by immobilizing the mAbs to Protein A-bound slides. With respect to binding to the higher-affinity V176 isoform, the dissociation constant (K_D_) of glycoforms bearing the galactose at the α6 antenna is twice as low as glycans lacking a galactose at this position (approximately 6 x 10^-9^ M *vs.* 11 x 10^-8^ M, respectively, **Figures 3C–F**). Similar to the ELISA results, minimal binding was observed with the GlcNAc-only palivizumab. As with our ELISA results, this is drastically stronger than steady state binding of commercially available palivizumab (10^-6^ M). A similar case is observed with the binding affinity of the various galactosylated afucosylated glycoforms to the low-binding F176 isoform, albeit at an approximate five-fold decrease of each glycoform.

**Figure 4:**
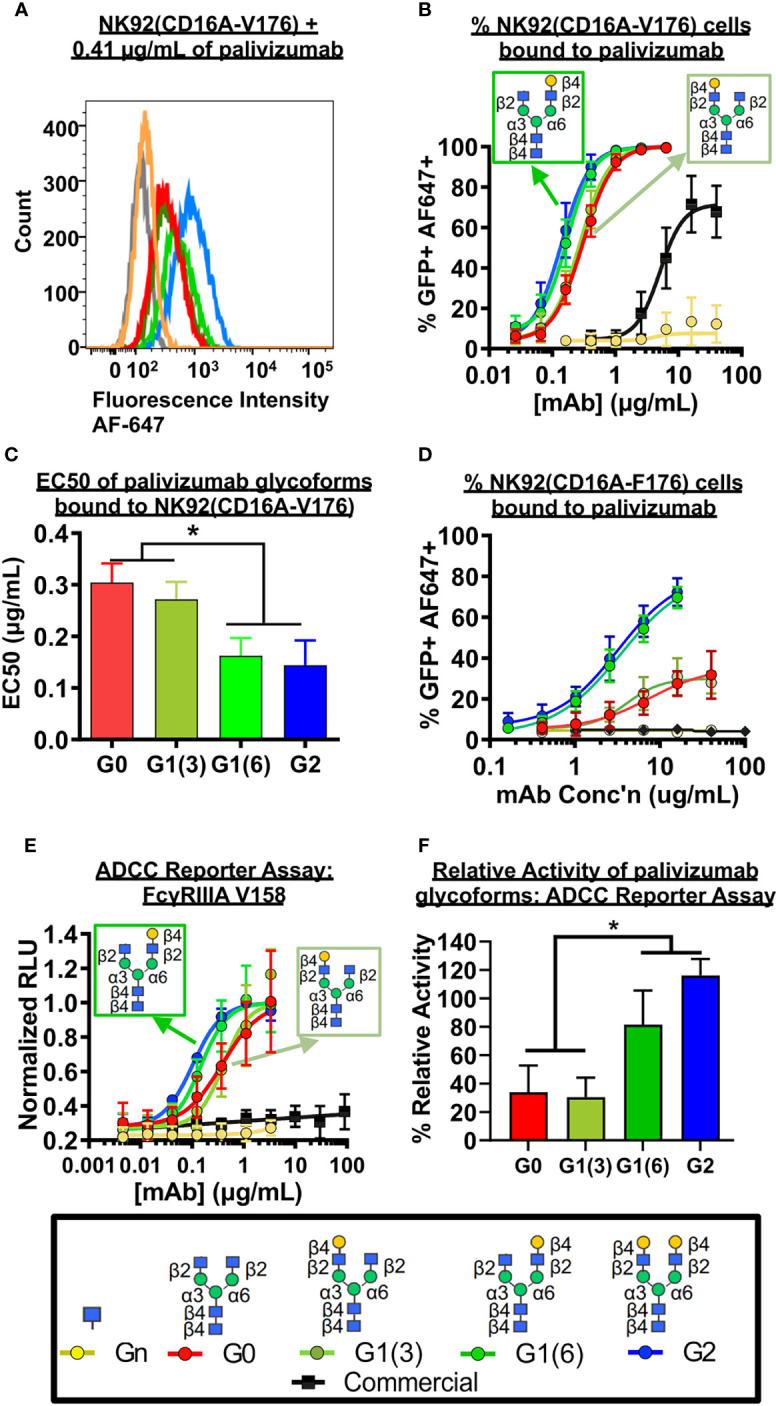
FcγRIIIa (CD16A) binding shows galactose on the α6 antenna significantly affects activity - Cell based assays. **(A-C)** NK92-CD16A expressing cells (V Variant) and **(D)** Jurkat T-cell luciferase reporter cell line (CD16A V Variant). **(A)** A representative flow cytometry histogram is shown of NK92-CD16A (V176) cells bound to 0.41 µg/mL of various palivizumab glycoforms that were detected using an anti-human IgG (F(ab’) _2_ –specific) conjugated to AlexaFluor(AF)-647. **(B)** Dose-response curve of % GFP+APC+ cells for each palivizumab glycoform analogue **(C)** EC 50 (µg/mL) shows that galactosylation in the α6 antenna result in approximately twice as low EC50 compared to glycoforms lacking galactosylation in this position. EC50s are calculated using GraphPad. *, p< 0.05, One-way ANOVA, Tukey *post-hoc* analysis. **(D)** Dose-response curve of % GFP+APC+ cells for each palivizumab glycoform analogue to NK92-GFP cells expressing the low-affinity CD16A F176 variant. **(E)** Dose-response curve and **(F)** % relative activity of palivizumab glycoforms in the ADCC reporter assay, where % relative activity is the ratio of the EC50 of G2-palivizumab to the EC50 of each sample in each biological replicate. For **(B-F)**, error bars show mean ± standard deviation (n=3 biological replicates); for **(B, D, E)** non-linear regression (4-PL) was used for curve-fitting in GraphPad; for **(C, F)** EC50 values were calculated and statistical analyses were performed using GraphPad. *, p< 0.05, One-way ANOVA, Tukey *post-hoc* analysis.

As coating recombinant proteins on to flat polystyrene surfaces can denature its conformation (40), and therefore skew binding affinity data, we next used more biologically-relevant model for FcγRIIIa binding, involving flow cytometry of a GFP-expressing natural killer T-cell line expressing the FcγRIIIa receptor (NK92-GFP-CD16A). NK92-GFP-CD16A cells bound with palivizumab at the Fc region was labeled with an AlexaFluor 647-conjugated anti-F(ab`)_2_ antibody fragment, and the percentage of GFP^+^AF647^+^ NK92 cells were analyzed by flow cytometry, **Figures 4A–C**. Similarly, to the SPR results, an approximate 2-fold enhancement in binding or EC-50 is detected for the glycoforms containing a galactose at the α6 antenna of afucosylated glycans (p < 0.05). Surprisingly, when using the lower affinity NK92-GFP-CD16A F176 variant, we observed a more pronounced difference between the glycoforms, **Figure 4D**. The presence of a terminal galactose residue on the α6 antenna results in a binding of approximately 70% of cells at 16 µg/mL, whereas palivizumab lacking this galactose residue resulted in a maximum binding of approximately 30%. Furthermore, the Endo S- and fucosidase-treated deglycosylated palivizumab and the commercially available fucosylated showed similar minimal binding to the low affinity CD16A F176 variant.

Next, we measured the ADCC activity of the afucosylated palivizumab glycoforms using RSV-infected Hep2 cells with the Jurkat T-cell luciferase reporter system (**Figures 4E, F**). Hep2 cells were infected with RSV overnight prior to incubation with palivizumab glycoforms and then the reporter cells were added. Consistent with the bioactivity to those of the aforementioned FcγRIIIA binding assays, increased ADCC reporter activity is observed in glycoforms comprising a terminal galactose residue at the α6 antenna (p < 0.05). In contrast, the commercial palivizumab yielded low ADCC activity, even at a 50-fold increase in concentration (100 µg/mL), **Figure 4E**.

### Characterization of glycan-Fc by NMR

**Figure 5:**
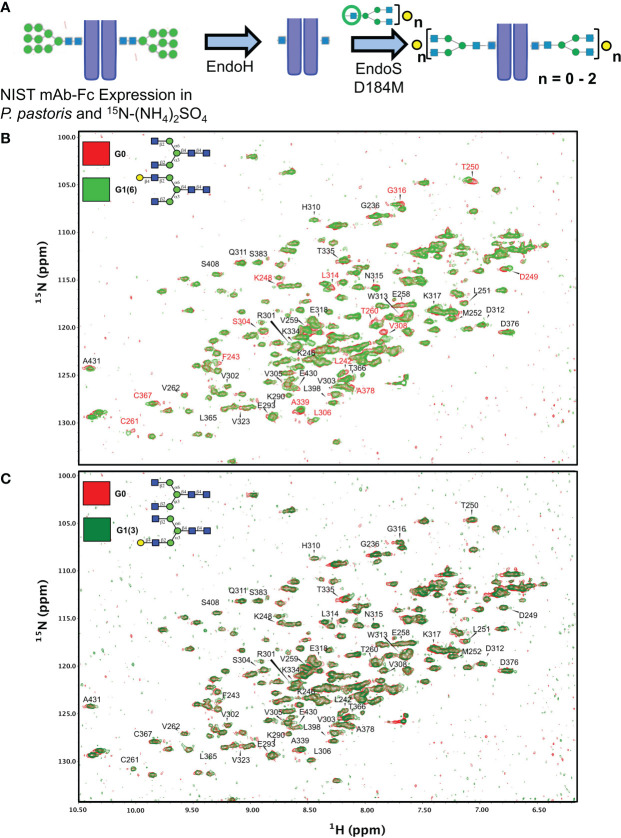
Synthetic scheme and NMR characterization of ^15^N-isotopically labelled Fc subunits with homogenous monogalactosylated glycoforms. **(A)** Synthetic scheme showing the remodeling of ^15^N-labelled Fc proteins expressed in *P. pastoris* and remodelled with sequential treatment with EndoH and EndoS D184M and purified glycan oxazolines. **(B, C)** Overlay of 2D-^1^H-^15^N NMR spectra of agalactosylated ^15^N-G0-NISTmAb-Fc (red) with **(B)** ^15^N-G1(6)-NISTmAb-Fc (bright green) and **(C)** ^15^N-G1(3)-NISTmAb-Fc (dark green). Peptide backbone amino acid residues are labelled, and Combined Chemical Shift Differences (CCSDs) are calculated for those labeled in red and are shown in **Table S1**.

The above observations points at the implication of the terminal galactose residue on the α6 antenna of the glycans. In order to ascertain the correctness of the glycoforms we prepared all four glycans on an isotopically labelled Fc fragment and applied NMR spectroscopy. As the NIST-mAb-Fc fragment shares the same amino acid sequence as the Fc fragment of palivizumab (41), we expressed this Fc fragment using *P. pastoris* yeast cells with (^15^NH_4_)_2_SO_4_ as the sole source of nitrogen, yielding a fragment that is over 98% labelled. Yeast expresses afucosylated high mannose glycans, and cleavage with Endo H produced the peptide bearing only a single GlcNAc residue at the N297 position. Treatment with EndoS D184M and the purified glycan oxazolines with variable galactosylation was performed, and completion was monitored by SDS PAGE (**Figure 5A**; **Supplementary Figure S6**).

Two dimensional (2D) ^1^H-^15^N-HSQC spectra were recorded for all four analogues along with its deglycosylated precursor (*i.e*. treated with EndoH). Since terminal galactose residues do not have proton-nitrogen pairs, chemical shift perturbations (CSPs) of the Fc protein backbone amides produced by the proximity of the galactose moiety were measured. The advantage of this approach is that the chemical shift of a single backbone amide pair is an absolute measurement of the magnetic environment surrounding this chemical group. In contrast, the intensity of a given NMR resonance can carry more variability due to many experimental factors (see Ghasriani et al. (42) for a more thorough discussion). In addition, binding or close proximity of a chemical entity such as a glycan perturbs the local magnetic environment of many amides. When the resonance assignment is known, this allows 2D-correlation spectra to identify binding sites, or presence and absence of nearby glycan moieties. The resonance assignments of the Fc fragment reported by Yagi et al. (43) were transposed onto the 2D-^1^H-^15^N-HSQC of ^15^N-G0-NISTmAb-Fc; despite the NIST mAb-Fc having a primary sequence that differs at residues 356-358 compared to the Fc fragment assigned by Yagi and coworkers (*i.e.* ^356^Asp-Glu-Leu^358^ *vs.* ^356^Glu-Glu-Met^358^, respectively), this difference in primary sequence is sufficiently distant from residues surrounding the glycans that assignment could be transposed.

We initially examined the 2D ^1^H-^15^N-HSQC NMR of the Endo H-deglycosylated ^15^N-Gn-NISTmAb-Fc with the agalactosylated G0 glycoform (**Supplementary Figure S7**), and observed numerous CSPs, indicative of the large effect that entire glycans have on the solution conformation of the Fc protein backbone compared to a single GlcNAc residue. Spectral overlays of monogalactosylated ^15^N-NISTmAb-Fc proteins with the agalactosylated ^15^N-G0-NISTmAb-Fc further revealed interesting features that confirmed the identities of these glycans and provide insight into our observations with the remodeled afucosylated palivizumab glycoforms (**Figures 5B, C**). Relative to the agalactosylated ^15^N-G0-NISTmAb-Fc, the presence of the terminal galactose in the α6 antenna (*i.e*. ^15^N-G1 (6)-NISTmAb-Fc) induces greater CSPs of the peptide backbone (amongst them are L242, F243, K248, D249, T250, T260, C261, S304, L306, V308, L314, G316, A339, C367, A378) (**Figure 5B**) compared to its presence on the α3 antenna alone (*i.e.* ^15^N-G1 (3)-NISTmAb-Fc, **Figure 5C**), indicating that galactosylation on the α6 antenna brings a galactose moiety in close proximity with the Fc protein. Next, we evaluated how galactosylation of each arm alone compared to digalactosylation on both arms (*i.e.* G2). Monogalactosylated ^15^N-G1 (6)-NISTmAb-Fc shows significant overlap and few CSPs compared to the digalactosylated ^15^N-G2-NISTmAb-Fc (**Supplementary Figure S7A**), whereas more CSPs are observed between the latter and the ^15^N-G1 (3)-NISTmAb-Fc (Supplemental **Figure S7B**), demonstrating that galactosylation at the α6 antenna alone is sufficient to impart a glycan conformation comparable to the digalactosylated glycoform. This may result from an interaction between the galactose on the α6 antenna and the protein backbone. However, our data (**Figure 5C**) suggest that galactosylation at the α3 antenna does not have these galactose-protein backbone interactions. As a complementary method to compare differences in chemical shifts between Fc-glycoforms, we calculated the combined chemical shift difference (CCSD, in ppm), a weighted value of the differences in chemical shifts in a 2D spectrum (**Supplemental Table 1**). The aforementioned 15 amino acid residues with the greatest CSPs between glycoforms with or without the terminal α6 antenna galactose showed greater CCSD values, whereas CCSDs of the same amino acid residues between glycoforms with the terminal α6 antenna galactose (*i.e*. G1 (6) and G2) are less than 0.02 ppm (with the exception of L242 and C261, which are 0.06 and 0.04 ppm, respectively). As a control peak that showed minimal CSPs between samples, D312 was used, which had CCSDs of less than 0.01 ppm, regardless of the terminal galactose position.

**Figure 6:**
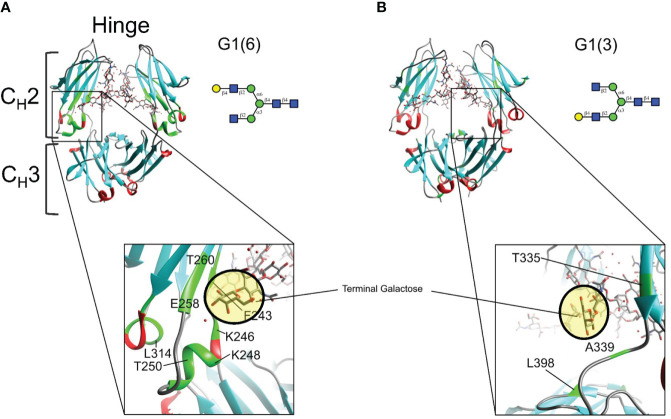
Mapping chemical shifts perturbations of Fc glycoproteins bearing a galactose residue at either the α3- or α6-antenna. **(A)** In the α6-antenna, the galactose is in close proximity with the protein backbone, whereas **(B)** in the α3-antenna, the galactose residue is exposed to the outer solvent. Residues that experiencing chemical shift perturbations are depicted in green. β-sheets are depicted in cyan, α-helix in red, and loops/turns are shown in gray. X-ray structure of glycosylated Fc (PDB ID: 4byh).

Mapping of twelve of these CSPs on the X-Ray structure (PDBID: 4byh) (44) highlight residues that are in close proximity with the galactose moiety of the α6 antenna (**Figure 6A**), which are located closer to the C_H_3 domain, while the fewer and weaker CSPs for the G1 (3) are consistent with a galactose moiety more exposed to the solvent, away from the protein backbone (**Figure 6B**). In this case, the very small CSPs limited the calculation of CCSD. Previous reports have shown that the latter antenna experiences greater mobility in solution (45), which would also contribute to smaller perturbations of chemical shifts.

## Discussion

Our method to generate asymmetrical mono-galactosylated glycans provides a facile alternative procedure to that previously reported (21, 39). Although monogalactosylated glycans at the α3 antenna only occurs at approximate 18% yield relative to digalactosylated glycans in sialoglycoprotein (SGP) extracted from inexpensive commercially available egg yolk, the ability to extract SGP at a relatively high yield enables its practicality. While the presence of a monogalactosylated species had been previously observed (34), it was neither further characterized nor isolated, as focus was on obtaining either the digalactosylated or completely agalactosylated glycans (33, 34). Furthermore, the selectivity of LacZ β1-4-galactosidase to highly favour the cleavage of the Gal-β1-4-GlcNAc linkage in the α3 antenna of digalactosylated biantennary complex glycans (38, 39), coupled with the ability of PGC-HPLC to separate isomeric glycans (22), our results demonstrate a feasible method to isolate purified asymmetrically galactosylated biantennary glycans.

Our FcγRIIIa binding experiments show that galactosylation of both antennae of afucosylated glycans (*i.e.* G2) of the anti-viral mAb palivizumab increases binding, corroborating a recent report with polyclonal serum-derived IgG (IVIG) used for anti-inflammation (12). Using chemoenzymatic glycan remodeling with asymmetric galactosylated biantennary glycans, herein we further refined the structural requirements for this binding; data from our various binding experiments consistently demonstrate that only the terminal galactose residue on the α6 antenna (*i.e.* G1 (6)) is required to impart this increased binding affinity. In its absence (*i.e*. G0 and G1 (3)), the binding affinity is comparable to the agalactosylated afucosylated (G0) glycan. Our KD values for both high- (V176) and low- (F176) affinity FcγRIIIA obtained by SPR analysis are within the same magnitude as those previously reported; for example, our digalactosylated afucosylated (G2) palivizumab glycoform had KD values of 5.9 ± 0.8 nM and 25.6 ± 1.3 nM with the high- and low-affinity receptors respectively, compared to 2 nM and 25 nM for G2-rituximab (18). However, it is worth noting that these KD values are lower than those obtained for Fc fragments (*i.e.* lacking the Fab region) comprising G2 (64 nM) (16). Our observed decrease in binding affinity by two-fold upon removal of the galactose on the α3-antenna (KD = ~ 6 nM for G1 (6) and G2 *vs* ~ 11-12 nM for G0 and G1 (3) for high-affinity FcγRIIIA, and ~ 25-31 nM for G1 (6) and G2 *vs* ~ 60 nM for G0 and G1 (3) for low-affinity FcγRIIIA) is similar to the 1.5- to 1.7-fold decrease previously reported between agalactosylated and digalactosylated afucosylated glycoforms (i.e. G0 vs G2) (16, 46). However, Wada et al. had previously reported a decrease in relative binding affinity by 3.6-fold, although KD values were not reported (20). Interestingly, the cell-based binding assay using NK92 cells showed a difference between the commercially-available high- and low-affinity CD16A variants. A two-fold difference in EC50 was observed with the high-affinity NK92-GFP-CD16A V176 variant, while only glycoforms with a galactose in the α6 antenna (*i.e.* G1 (6) and G2) showed any appreciable binding to the low-affinity NK92-GFP-CD16A F176 variant.

While the increased FcγRIIIa binding in monogalactosylated at the α6 antenna was recently shown in fucosylated glycans (21) in the anti-inflammatory drug rituximab, we now demonstrate that this is also applicable to afucosylated glycans and in the context of an anti-viral mAb that binds different targets than rituximab. Commercial palivizumab (Synagis), which is predominantly core fucosylated, is generally considered to be a prophylactic mAb whose mechanism of action is acting as a neutralizing agent, rather than by actively destroying infected cells (47, 48), and has been shown to have relatively low ADCC activity (48). Our ADCC reporter assay results using RSV-infected Hep2 cells corroborate the low activity of commercially-available palivizumab, even at 90 µg/mL (**Figure 4E**, black data points) (48). A previous report had shown that palivizumab expressed in *N. benthamiana* changed the glycoprofile from predominantly fucosylated glycans into one that comprised 79% agalactosylated, afucosylated (G0) glycans, and 17% of high mannosylated (Man 7/9) glycans (25). Our data here shows the synthesis of a higher purity of glycans with varying degrees of galactosylation (97%), with 3% high mannosylated (Man 5) glycan, and that upon galactosylation of the α6 antenna, we can further increase palivizumab binding to FcγRIIIa and ADCC activity by approximately two-fold compared to the agalactosylated afucosylated palivizumab (*i.e.* G0) glycoform. However, it is important to note that our comparison between the afucosylated glycoforms and commercial product (that is mostly fucosylated) is consistent with previous literature that removal of the core fucose residue remains a dominant feature to increase FcγRIIIA binding. Rather, our results provide further corroborating evidence with more recent studies that galactosylation further increases ADCC *via* FcγRIIIA binding (12, 21, 49) and is in contrast to previous studies that showed galactosylation had no effect (11, 13, 50). During the biosynthesis of complex *N*-glycans in glycoproteins, β-1,4-galactosyltransferase is involved in attaching the galactose residue to the terminal GlcNAc residue in complex *N*-glycans; Ramasamy et al. previously demonstrated that the human β-1,4 galactosytransferase exhibits selectivity towards the α6 antenna at lower glycan concentrations, while the α3 antenna is favoured at higher glycan concentrations (51). As this key enzyme can have opposing selectivity as a function of glycan concentration, this further emphasizes the need to carefully control conditions during manufacturing and production of glycoprotein-based biotherapeutics, and together with our data that shows the importance of galactose location within the Fc-glycan on FcγRIIIA binding, the proper characterization of glycan isomeric structures needs to be considered to properly evaluate the biological properties of monoclonal antibodies (51).

Previous data reported that soluble FcγRIIIA receptors bound to Fc proteins near the hinge region of the C_H_2 domain (via hydrogen bonds at residues G236, G237, P238, S239, D265, Y296, as well as glycan-glycan interactions with the N162 glycan of sFcγRIIIA), and show that the terminal galactose moiety of the α6-antenna is far from any Fc- FcγRIIIa contacts (PDBIDs: A3Y4 (8), 3SGJ (7), 5D6D (52)). Therefore, the correlation between the enhancement of receptor affinity and biological activity with the presence of the terminal galactose residue at the α6-antenna of the glycan can be *a priori* puzzling. However, several reports suggest that glycan dynamics also play an important role in receptor affinity. Barb and Prestegard have shown using predominantly fucosylated and ^13^C-labeled galactose on the α6 antenna, that this latter carbohydrate residue has broader resonance lines indicative of slower mobility, while the α3 antenna has increased mobility (45). Mutations to the Fc peptide backbone that increase galactose mobility of the α6 antenna are correlated to a 20- to 50-fold decrease of FcγRIIIA receptor affinity (53, 54). The chemoenzymatic strategy used herein to generate defined afucosylated, monogalactosylated glycans on a uniformly ^15^N-labeled Fc fragment brings further support to the role of glycan dynamics. Two-dimensional NMR spectra of the ^15^N-labeled Fc protein monogalactosylated at the α6 antenna show several chemical shift perturbations (CSPs) compared to the agalactosylated glycoform, indicating that the α6 antenna is in close proximity to the protein backbone and is consistent with reduced mobility; our use of ^15^N-labeled Fc with defined monogalactosylation at the α6-antenna shows that this occurs independently of galactosylation at the α3 antenna. Conversely, the spectrum of ^15^N-labeled Fc comprising monogalactosylation at the α3 antenna show small and weak CSPs which is consistent with a solvent-exposed galactose with greater mobility, and occurs independently of the presence of an α6-antennal galactose. It was not useful to measure CCSD for the perturbations observed in G1 (3) relative to G0 since the changes that are observed are either a change of peak shape (narrowing or broadening) or simply disappearance, depending of the frequency of the change in the local magnetic environment. The only residue that shows a clear CSP is T335 with a CCSD of 0.05 ppm where the peak shape has also changed (narrowing). The fact that the spectral overlay of G0 and G1 (3) is mainly showing changes in peak shape is consistent with a mobile galactose moiety that is mainly exposed to the solvent with little to no interactions with the protein backbone. The presence of the afucosylated α6-antennal galactose causes CSPs in a region closer to the C_H_3 domain (*i.e.* F243, K248, D249, T250, T260, C261), and are in line with a previous study showing that residues F241 and F243 are in close proximity with this galactose in fucosylated digalactosylated glycans (G2F), and mutation of these residues increase glycan mobility and reduce FcγRIIIA receptor affinity (53). It is therefore plausible that the presence of an α6-antennal galactose (*i.e.* G1 (6) or G2) reduces mobility of afucosylated Fc-glycans by interacting with the Fc protein backbone, which enhances FcγRIIIA receptor affinity, similarly to previous reports with fucosylated glycans (53). However, the effect of glycan-glycan interactions involving the α6-antenna terminal galactose residue of a full mAb and the *N*-glycans of the FcγRIIIA receptor cannot be ruled out, although previous X-Ray work did not identify these interactions using truncated Fc proteins (8).

While afucosylation remains the predominant factor for FcγRIIIa binding and ADCC activity, this two-fold increase in activity based on the location of the terminal galactose residue highlights the importance to carefully characterize glycan structures in biotherapeutics. From a regulatory perspective, depending on the mechanism of action, this may warrant attention when evaluating physicochemical data to assess batch-to-batch variability and comparing biosimilars to existing innovator mAb products.

## Materials and methods

### Glycan isolation, purification and oxazoline formation

Sialoglycoprotein (SGP) was isolated from commercially available egg yolk as previously described (33). Glycans were released from SGP using EndoS (New England Biolabs, Cat #P0741) containing a chitin-binding domain (CBD). EndoS-CBD was immobilized to chitin resin (New England Biolabs, Cat # S6651) by incubating 10 uL EndoS-CBD with 1.0 mL chitin resin slurry (0.5 mg/mL) overnight at 4°C. The slurry was mixed well using wide-bore pipette tips and transferred to a solution of SGP (10 mg/mL containing GlycoBuffer 1 (New England Biolabs, Cat # B1727S). 5 µL α2-3,6,8 neuraminidase (NEB, Cat #P0720) was then added to desialylate glycans. The reaction was incubated at 37°C with gentle agitation for 3 days, and monitored by HPAEC-PAD. For HPAEC-PAD analysis, a Dionex CarboPac PA200 IC column (3 mm x 250 mm, 5.5 µm particle size, Cat # 062896) was used, with a gradient of 20%/0%/80% to 60%/20%/20% of MP-A/MP-B/MP-C (MP-A: 200 mM NaOH; MP-B: 150 mM NaOAc in 200 mM NaOH; MP-C: MQ H_2_O) over 10 min at 30°C was used to monitor glycan products. Following reaction completion, resin was removed by passing through a solid phase extraction tube and rinsed with MQ water. The reaction lyophilized, resuspended in MQ water and filtered through a 0.22 µm PVDF filter before it was purified by PGC-HPLC to isolate G1 (3)T and G2T glycans (HyperCarb PGC column (Thermo Fisher Cat # 35005-159070A, 10 cm x 150 cm, 5 µm particle size). Following an initial mobile phase composition of 1% MP-D: MP-E (MP-D: 95% acetonitrile in MQ H_2_O containing 0.1% TFA; MP-E: MQ H_2_O containing 0.1% TFA), a gradient of 6.0% to 13.7% MP-D: MP-E was used from 1.0 min to 42.0 min at 5 mL/min, at 40°C. Glycans were detected at 214 nm using a UV detector, collected using a fraction collector, analyzed by HPAEC-PAD prior to fraction combination, and lyophilized. Impure samples were re-purified as above. The β- and α- anomers of G1 (3)T eluted at approximately 23.0 and 28.0 min, while the anomers of G2T eluted at approximately 32.0 min and 37.0 min.

To prepare glycans containing zero or one terminal galactose residue (at the α6 antenna only) (G0T, G1 (6)T), G2T was diluted to 5 mg/mL (1.0 mL) with 150 µL of KCl (500 mM), 150 µL of a buffer containing 500 mM sodium acetate and 50 mM CaCl_2_ (pH 5.7), and 4.55 mL MQ water. 150 uL β-1,4 galactosidase (Sigma, Cat# 10105031001) was then added, and the reaction was incubated at 30°C overnight. The reaction was monitored by HPAEC-PAD and purified by PGC-HPLC as above. The β- and α-anomers of G0T eluted at approximately 17.0 and 22.0 min, while the anomers of G1 (6)T eluted at approximately 26.0 min and 31.5 min.

With each purified glycan in hand, each was converted to their respective oxazoline intermediates by reaction with 2-chloro-1,3-dimethylimidazolinium chloride (DMC, Sigma, Cat# 529249-25G) and trimethylamine (TEA, Fisher, Cat #0484-100) at 4°C, and purified using 0.1% ammonium hydroxide in a column packed with G25 resin (Fisher, Cat # 45-002-048). Collected fractions were monitored by HPAEC-PAD, and those containing purified oxazolines were combined, lyophilized and stored at -80°C until use.

### Monoclonal antibody transglycosylation

Methods were adapted from previously reported work (17, 32), with the following modifications. Palivizumab was purchased from McKesson Specialty Pharmacy (Cat #02438272), and the required amount of antibody was diluted to 10 mg/mL in PBS. For the scale of 1 mg (in 100 µL), palivizumab was first digested with EndoS D233Q (32) by adding 50 µL (2.0 mg/mL in PBS), followed by the addition of 100 µL fucosidase (GH29, CedarLane, Cat# CZ05662) that has been previously removed of glycerol. The reaction was incubated overnight at 37°C, and completion was monitored by SDS-PAGE. With the same EndoS D233Q remaining in the crude deglycosylated reaction mixture, deglycosylated palivizumab was passed through Amicon centrifugal filters (30 kDa MWCO, Sigma, Cat# UFC5030) to buffer exchange into cold transglycosylation buffer (50 mM Tris, pH 7.3) and concentrated back to 10 mg/mL. The solution was placed in a 30°C heating block, and glycan oxazoline (6 µL, 400 nM) was prepared and kept on ice. 1 µL of the oxazoline solution was added to the deglycosylated palivizumab containing EndoS D233Q every 5 minutes for a total of 6 additions. After a total reaction time of 30 mins, the reaction was quenched by dilution with 500µL of cold Protein A binding buffer buffer (100 mM Na_2_PO_4_, 100 mM NaCl, pH 8.0). To the solution, 250 µL rinsed Protein A resin slurry (ThermoFisher Scientific, Cat # 22810) was added and incubated at 4°C with gentle rocking for 5 min. The solution was then passed through a solid phase extraction tube and washed with cold Protein A binding buffer (0.75 mL x 2), followed by a 50 mM sucrose solution in PBS (1.0 mL x 2) and then cold Protein A binding buffer (0.75 mL x 2). Palivizumab was then eluted from the Protein A column using 50 mM sodium citrate buffer (pH 3.5), and neutralized with 200 µL of Protein A binding buffer. Eluted palivizumab was monitored using SDS PAGE and fractions were combined and passed through Amicon centrifugal filters (30 kDa MWCO) to buffer exchange with Citrate Buffered Saline (130 mM NaCl, 20mM sodium citrate, pH 5.7) and concentrate palivizumab. Solutions were stored at 4°C at approximately 0.3 – 1.0 mg/mL, as quantified by nanodrop (at Absorbance of 280 nm).

### Monoclonal antibody characterization

Reaction progress for glycan remodeling of palivizumab was monitored and characterized using reducing SDS PAGE and intact protein mass spectrometry. For reducing SDS PAGE, samples reduced in β-mercaptoethanol for 5 mins at 100°C, followed by loading onto 10% polyacrylamide gels which were then run at 200 V for 70 mins. To ensure accurate analysis of band migration at various glycosylation states, a lane was run comprising both starting material and final product within the same lane.

For mass spectrometry, samples were analyzed with an Orbitrap Fusion Lumos Tribrid Mass Spectrometer coupled to an Easy-nLC 1200 (Thermo Scientific) that was calibrated by infusion prior to analysis with a mixture of caffeine, MRFA, and Ultramark 1621. 0.5 µL of each sample was analyzed by loading onto a C4 Pepmap300 PS-DVB trap column (300A x 5µm x 5mm) and desalting with 0.1% formic acid in water (solvent A) before separating on a PepSwift Monolithic PS-DVB analytical column (200 µm x 25 cm) at 50°C. Chromatographic separation was achieved at a flow rate of 1 µl/min over 25 min in five linear steps as follows (solvent B was 0.1% formic acid in 80% acetonitrile): initial, 0% B; 5 min, 50% B; 10 min, 50% B; 20 min, 90% B; 25 min, 90% B. The eluting proteins were analyzed in Intact Protein mode with Orbitrap MS at 15000 resolutions, from 500-4000 m/z with auto maximum injection time, normalized AGC target of 50%, and 10 microscans. BiopharmaFinder3.0 (Thermo) was used to process the data. Protein deconvolution was performed with the ReSpect algorithm, using mass output of 130 – 160 kDa, Model Mass Range of 145000 – 150000, Charge State Range 40-80.

Following palivizumab transglycosylation reactions, remodelled glycans were characterized using PNGaseF (New England BIolabs, Cat # P0704) to release glycans, followed by HPAEC-PAD analysis. For HPAEC-PAD analysis, a Dionex IC-3000 instrument was used, equipped with a Dionex CarboPac PA200 IC column (3 mm x 250 mm, 5.5 µm particle size, Cat # 062896) and a Gold Standard PAD waveform with an AgCl electrode. For glycoprofile analyses, a flow rate of 0.4 mL/min, and the following gradient was used: 20%/0%/80% to 25%/5%/70% of MP-A/MP-B/MP-C over 25 mins at 30°C, followed by an increase to 0/80/20 over the next 3 mins, and then re-equilibration to 20/0/80 over the next 10 min.

### FcγRIIIA binding assay by ELISA

Recombinant human FcγRIIIA proteins were immobilized onto MaxiSorp plates (ThermoFisher, Cat# 349454) overnight at 4°C, at 100 µL of 0.33 µg/mL for FcγRIIIA V176 (high affinity, R&D Systems, Cat# 4325-FC-050), or at 0.67 µg/mL for FcγRIIIA V176F (low affinity, R&D, Cat# 8894-FC-050). Following washing with 0.05% Tween 20 in PBS pH 7.4 (PBS-T) and blocking with 1% bovine serum albumin (Sigma, Cat # A3059-100G) in PBS-T, each palivizumab glycoform was serially diluted 2-fold in 1% BSA/PBS-T and 100 µL was added to each well. Plates were gently rocked for 20 min at room temperature, followed by incubation at 4°C overnight. The next day, wells were thoroughly washed with PBS-T, followed by addition of goat anti-Human IgG F(ab’)2 Secondary Antibody-HRP (100 µL, 1/500 dilution in 1% BSA/PBS-T, ThermoFisher, Cat # PI31482). The plate was then gently rocked at room temperature in the dark for 45 mins, followed by thorough washing with PBS-T. 50 µL of Ultra-TMB (ThermoFisher, PI34028) was added, and the plate was gently rocked in the dark for 10 mins, before quenching with 50 µL of 2M H_2_SO_4_. Plates were immediately read on a BioTek Plate reader at 450 nm.

### FcγRIIIA binding assay by surface plasmon resonance analysis

Samples were performed by the National Research Council Human Health Therapeutics Research Centre. Experiments were carried out using a Biacore T200 SPR instrument (Cytiva Inc., Marlborough, MA) at 25°C with a running buffer comprising PBS with 0.05% Tween 20 and 3.4 mM EDTA. Protein A (GenScript Inc., Piscataway, NJ) was immobilized onto two sequential CM-5 sensorchip surfaces at 2000 RUs each using standard amine coupling within the Biacore Immobilization Wizard. Palivizumab analogues were captured onto the second protein A surface at 2.5 µg/mL for 30 s, with the first protein A surface remaining as a blank control surface. FcγRIIIA CD16A isoforms (high binding (V) and low binding (F) variants) were produced and purified by the National Research Council (Canada), as described previously (55). Sensorgrams for FcγRIIIA binding to captured palivizumab were generated using a single-cycle kinetics method, which consist of five FcγRIIIA injections (25 µL/min injection time; 40 s contact time) of increasing concentrations between 2.3 nM and 0.56 µM were used, followed by a single dissociation phase of 120 s for each interaction. The protein A surfaces were regenerated with a 30 s pulse of 10 mM glycine pH 1.5 between each capture and injection cycle. The FcγRIIIa sensorgrams were double referenced and fit to a 1:1 binding model for kinetic determination with affinity (KD) being based on the ratio of the calculated rate constants. Each binding experiment was repeated three times for each analogue.

### FcγRIIIA binding assay by flow cytometry

NK92-GFP-CD16A cell lines were purchased from American Type Culture Collection (ATCC, V Variant: Cat # PTA-8836; F variant: Cat # PTA-8837) and cultured according to manufacturer protocols. 2.5-fold serial dilutions of palivizumab glycoforms were prepared (80 µL per well) in cold deep-well 96-well plates and kept at 4°C, using cold 1% bovine serum albumin (Sigma, Cat # A3059-100G) in PBS as the diluent. To prepare cells for palivizumab-FcγRIIIa binding experiments, NK92 were harvested, and washed with 1% BSA/PBS. Following re-suspension in 1% BSA/PBS with 2 mM EDTA, cells were counted using a haemocytometer. NK92 cells were mixed thoroughly, diluted to 0.625 x 10^6^ cells/mL, and 40 µL was added into each well and mixed gently with a pipette, followed by gentle rocking at 4°C for 30 mins. Plates were then washed with 0.5 mL of cold 1% BSA/PBS, centrifuged at 1700 x rpm (7 min) and supernatant removed.

To each well, 150 µL of a 1/300 dilution of Alexa Fluor^®^ 647-conjugated Goat Anti-Human IgG - F(ab’)_2_ specific (AffiniPure F(ab’)_2_ Fragment) (Jackson, Cat # 109-606-097) was then added, and incubated with gentle rocking at 4°C for 30 mins. As a positive control, APC-CD16 Ab, anti-human, REA423 (Miltenyi Biotec, Cat # 130-113-951) at 1/50 dilution was used. As a negative control, the aforementioned AlexaFluor-647 AffiniPure F(ab’’)_2_ Fragment was added, in the absence of any palivizumab. Then cells were washed with 1 mL of cold 1% BSA/PBS with 2 mM EDTA and centrifuged as above, followed by re-suspension in 135 µL of 1% BSA/PBS, and transferred into a shallow 96-well plate containing 45 µL of 4% PFA/PBS. Shallow plates were incubated with gentle rocking at room temperature for 30 min, followed by centrifugation at 100 xg (1 min) and removal of 140 µL of supernatant. Cell pellets were re-suspended in 80 µL of 0.4 M glycine (Sigma, Cat# G8898) in PBS. For each biological replicate (n=3 total), technical duplicates were performed.

Plates were then read using a BD LSR II Flow cytometer (FACS Diva, v 8.0.1), equipped with a multi-well plate reader and the following laser/filter sets: Blue laser 488nm (20mW) with GFP filter set: 505 Long Pass 525/50 Band Pass and SSC filter set: 488/10 Band Pass and FSC; Red Laser 640nm (40mW) with APC/AF647 filter set: 660/20 Band Pass. Voltages were optimized in initial experiments and saved as application settings. Application settings were used in FACS DIVA for all voltages in all subsequent experiments to keep measurements consistent between experiments. Data was analyzed using FlowJo (v 10.8.1), and the gating hierarchy is as follows: (i) FSC-A vs SSC-a, (ii) FSC-H vs HSC-W, (iii) SSC-H vs SSC-W, (iv) GFP+, (v) AF647+/-.

### ADCC reporter assay with palivizumab glycoform analogues

ADCC assays were performed using the ADCC Reporter Bioassay, V Variant (Promega, Cat #G7010). To adapt it to the RSV model, Hep2 cells were first cultured in a 96-well plate at a cell density of 2.0 x 10^4^ cells/well in 100 µL of DMEM media with 10% low IgG FBS (Fisher, Cat# SH3089802), and incubated at 37°C for 24 h. The next day, cells were infected with 100µL/well of RSV in DMEM containing 10% low IgG FBS, at a MOI of 5, with followed by incubation at 37°C for 24 h. RSV was removed, and wells were washed with PBS (200 µL). 25 µL of RPMI 1640 containing 10% low IgG FBS was immediately added to each well. Solutions of palivizumab glycoforms were diluted in RPMI 1640 with 10% low IgG FBS and 25 µL were added into each well, and incubated for 37°C for 30 min. A vial of effector cells was thawed according to manufacturer protocols, and 25 µL were added into each well and incubated for 37°C for 4 h 45 min. 75 µL Bio-Glo luciferase reagent was then added to each well and the plate was immediately read on a BioTek plate reader.

### Synthesis of ^15^N-isotopically-labeled Fc

A starting culture in 50 mL yeast nitrogen based (YNB) media, containing 3.4 g/L of YNB powder without amino acids (Sigma), 100 mM KH_2_PO_4_ at pH 6.0, 10 g/L of ^15^N-ammonium sulfate, 10 g/L D-glucose, 20 µg/L of biotin, was inoculated with a single colony of Pichia X33-NIST-mAb-Fc and incubated at 29°C until an optical dispersion (OD_600_) of 0.4 is obtained. An aliquot of 22 mL of this culture was used to inoculate a 450 mL of YNB growth media and incubated at 29°C until an optical dispersion (OD_600_) of 6.6 is obtained. Optical density is accurately measured with a 1:10 dilution of an aliquot from the culture with water. In order to remove unspent glucose that would inhibit protein synthesis, cells were harvested by centrifugation @ 3000 X g for 10 minutes then re-suspended in i-wash media (same composition to YNB, but without glucose) and incubated at 25°C for 1 h. Cells were then centrifuged and re-suspended in 25 mL in wash media, and sufficient amount of the slurry (18 mL) to obtain an OD_600_ of 1.0 was used to inoculate 2 X 1 L of induction media (same composition to YNB growth media with the glucose replaced by 10 mL/L of methanol). Protein induction was carried out for 72 hours at 29°C and 10 mL of methanol 5% were added per liter at 24 h and 48 h post-induction. Cells were separated from media containing ^15^N-NIST-mAb-Fc by centrifugation at 3000 X g for 10 minutes at 4 °C. To inhibit proteolytic cleavage of the target protein, benzamidine (5mM) and EDTA (0.5 mM) were added, then the pH of the media at 5.84 was adjusted to 7.3 with the addition of potassium hydroxide (10 N). After pH adjustment, a white precipitate formed. After 30 minutes, the precipitate was separated by centrifugation.

The target protein was purified by affinity chromatography by loading the solution on a 15 mL protein A column at 1 mL/min. Elution of the protein was carried out with 0.1 M glycine-HCl at pH 3.0 after a wash of the resin with 1x PBS (with protease inhibitors). Fractions containing the ^15^N-NIST-mAb-Fc were dialysed against 50 mM sodium citrate pH 5.5 prior to glycan removal. The high mannose rich glycan was cleaved using 20000 units of Endo H (New England Biolabs, Cat # P0702S) per 5 mg of protein for 2 hours at 37°C. The reaction mixture was then purified using the Protein A affinity chromatography, then fractions containing the target protein were dialyzed against 50 mM sodium acetate pH 5.0.

### NMR spectroscopy

Data acquisition was acquired as previously reported (56). All NMR samples contained the appropriate fragments at approximately 15 μM protein concentration in 20 mM sodium phosphate at pH 6.0 and 5% v/v deuterium oxide for field frequency lock purposes. Spectra were recorded on Bruker AVANCE III-HD 700 MHz spectrometers equipped with a 1.7 mm TCI cryoprobe. All NMR data were collected at 50 °C in 1.7 mm capillary tubes containing 40 µL of sample. Proton-nitrogen correlation spectra were collected using the phase-sensitive sofast-hmqc pulse sequence from the Bruker library (sfhmqcf3gpph) with the following parameters: the pc9 pulse with a 120 ° excitation was increased to 2210 μs, corresponding to a 3400 Hz bandwidth, (the pc9 pulse has bandwidth factor of 7.512) and centered at 8.4 ppm, 8096 transients per FID were collected to produce a data matrix of 512 × 64 complex points for a total acquisition time of 108 h. The nitrogen dimension used a spectral window of 35 ppm that was centered at 117 ppm (corresponding to a 26 ms acquisition time for the nitrogen dimension). The inter scan delays was 0.3 s. All NMR spectra were processed with NMRPipe and viewed using NMRView (57, 58). Structural models were visualized using chimera (59). Combined Chemical Shift Difference (CCSD) was calculated based on the following equation:

where α=0.1 and δH and δN are differences in chemical shifts in ppm for ^1^H and ^15^N with respect to a reference cross peak (42).


CCSD=√ (0.5  *  [ (δH) 2 + (α * δN) 2 ] )

### Statistical analysis

All statistical analyses were performed using GraphPad Prism version 9.0.0 (GraphPad Software, San Diego, CA, USA). Non-linear regression (variable slope, four parameters) was used for curve-fitting, and differences amongst two treatments were assessed using one-way ANOVA with Tukey *post hoc* tests to identify statistical differences. An α level of 0.05 was set as the criterion for statistical significance. Graphs are annotated with p values represented as *p ≤ 0.05, **p ≤ 0.01, ***p ≤ 0.001, ****p ≤ 0.0001. All data are presented as mean + standard deviation.

## Data availability statement

Mass Spectrometry data presented in the study are deposited in the FigShare repository, doi: 10.6084/m9.figshare.20171174.v1.

## Author contributions

GH synthesized glycans, performed glycan remodeling on monoclonal antibodies/^15^N-NISTmAb-Fc proteins, and data analysis. LT performed binding and cell-based assays. GG expressed ^15^N-NISTmAb-Fc proteins. AS performed flow cytometry experiments and analyzed data. XL conceptualized experiments provided resources for cell culture experiments and edited manuscript. YA performed NMR experiments, experimental conceptualization, data interpretation, wrote and edited manuscript. RT conceptualized experiments, synthesized glycans, performed cell-based assays, data analysis and interpretation, wrote and edited manuscript. All authors contributed to the article and approved the submitted version.

## Funding

This work was funded by the Government of Canada.

## Acknowledgments

We thank Yi-Min She, Lisa Walrond and Marybeth Creskey of the Mass Spectrometry lab at the Regulatory Research Division at Health Canada for performing mass spectrometry sample analyses. We thank Drs. Jessie Lavoie, Michael Johnston, Simon Sauvé and Michael Rosu-Myles for critical review of this manuscript and thoughtful comments.

## Conflict of interest

The authors declare that the research was conducted in the absence of any commercial or financial relationships that could be construed as a potential conflict of interest.

## Publisher’s note

All claims expressed in this article are solely those of the authors and do not necessarily represent those of their affiliated organizations, or those of the publisher, the editors and the reviewers. Any product that may be evaluated in this article, or claim that may be made by its manufacturer, is not guaranteed or endorsed by the publisher.

## Supplementary material

Supplementary Figure 1
*N*-glycan characterization of palivizumab. **(A)** HPAEC-PAD chromatogram of commercially-available palivziumab and **(B)** glycan analysis of palivizumab obtained commercially, compared to those reported in literature (25) **(C)** Intact mass spectrometry analysis of commercially available palivizumab shows the presence of various glycoforms on the same mAb (*i.e*. on each heavy chain of the mAb dimer). **(D)** Theoretical and observed intact mass values (Da) and mass error (ppm) of various glycoforms in commercial palivizumab.

Supplementary Figure 2 Mass spectrometry characterization of branch-specific truncated monogalactosylated isomers **(A)** G1(3)T and **(B)** G1(6)T, obtained following purification by PGC-LC-HPLC. MS/MS spectra are shown at the doubly charged ions of m/z 638.7374 and 638.7375 for **(A)** and **(B)**, respectively. Cleavage of the α3 antenna between the GlcNAc-β1-2-Man linkage produces a relatively higher abundance of the cleaved and its complementary glycan fragment compared to cleavage of the α6 antenna.

Supplementary Figure 3 Characterization of defucosylated palivizumab analogues with varying degrees of galactosylation. **(A)** SDS-PAGE analysis shows the shift in bands between starting material (deglycosylated) and completed reaction (glycosylated). **(B-G)** Deconvoluted intact mass spectrometry spectra shows the presence of palivizumab **(B)** following deglycosylation with EndoS, **(C)** Fucosidase GH29, then transglycosylation with EndoS D233Q and purified oxazoline glycans to form homogeneous palivizumabs comprising **(D)** agalactosylated G0, **(E)** monogalactosylation at the α3 antenna (G1(3)), (**F**) monogalactosylation at the α6 antenna (G1(6)), or**(G)** digalactosylated G2. **(H)** Theoretical and observed intact mass values (Da) and mass error (ppm) of various palivizumab glycoform intermediates and final products following enzymatic transglycosylation.

Supplementary Figure 4 HPAEC-PAD chromatogram of PNGase-F-released *N*-glycans from remodeled palivizumab. Un-remodelled Man5 glycans were 3% for each analogue, indicating that enzymatic glycosylation is consistent for all remodelled analogues.

Supplementary Figure 5 Characterization of transglycosylated ^15^N-NISTmAb-Fc with homogeneous glycoforms. Reducing SDS-PAGE gels show the shift in band migration of transglycosylated isotopically ^15^N-NISTmAb-Fc (with G0, G1(3), G1(6), or G2) compared to starting material comprising only a single GlcNAc residue on each monomer. Lanes showing two bands contain both starting material and the transglycosylated Fc product to ensure these are two discreet bands.

Supplementary Figure 6 Overlay of 2D ^1^H-^15^N NMR spectra of EndoH-treated ^15^N-Gn-NISTmAb-Fc (orange) and remodelled ^15^N-G0-NISTmAb-Fc (red).

Supplementary Figure 7 Overlay of 2D ^1^H-^15^N NMR spectra of digalactosylated ^15^N-G2-NISTmAb-Fc (blue) with **(A)**. monogalactosylated remodelled ^15^N-G1(6)-NISTmAb-Fc (green), and **(B)** ^15^N-G1(3)-NISTmAb-Fc (dark green). Peptide backbone amino acid residues that show chemical shift perturbations (CSPs) are labelled and highlighted with a red box, while examples of other residues that show little to no CSPs are labelled as black text only without a red box. Compared to the digalactosylated protein, more chemical shift perturbations are observed with the monogalactosylation at the α3 antenna **(B)** compared to the α6 antenna **(A)**.

Supplementary Table 1
^1^H and ^15^N chemical shifts (in ppm) and Combined Chemical Shift Difference (CCSD, in ppm) of amino acid residues that show Chemical Shift Perturbations (CSPs) between agalactosylated afucosylated Fc glycoform (G0) with glycoforms bearing a terminal galactose residue at the α6 antenna. Low CCSD values are observed amongst afucosylated glycoforms with a terminal galactose residue at the α6 antenna (i.e. G1(6) vs G2). D312 is used as a reference for a residue that showed low CCSD in all analogues.
