# Supplementary material for: Specific location of galactosylation in an afucosylated antiviral monoclonal antibody affects its FcγRIIIA binding affinity
Source: Front Immunol. 2022 Oct 12;13:972168. doi: 10.3389/fimmu.2022.972168 (PMC9596277; doi:10.3389/fimmu.2022.972168)
Supplement: Supplementary Figure 1 — N-glycan characterization of palivizumab. (A) HPAEC-PAD chromatogram of commercially-available palivziumab and (B) glycan analysis of palivizumab obtained commercially, compared to those reported in literature (25) (C) Intact mass spectrometry analysis of commercially available palivizumab shows the presence of various glycoforms on the same mAb (i.e. on each heavy chain of the mAb dimer). (D) Theoretical and observed intact mass values (Da) and mass error (ppm) of various glycoforms in commercial palivizumab. [file DataSheet_1.pdf]

Specific location of galactosylation in an afucosylated antiviral monoclonal antibody affects its FcγRIIIA binding affinity

Grayson Hatfield, Lioudmila Tepliakova, Genevieve Gingras, Andrew Stalker, Sean Li, Yves Aubin, Roger Y. Tam

A

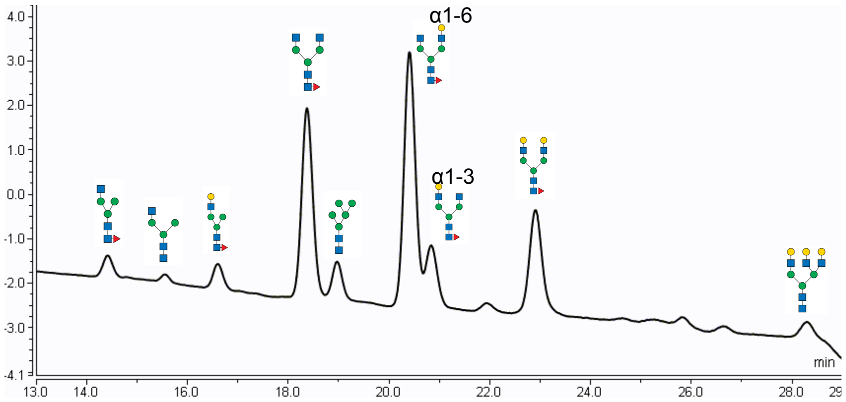

B

Palivizumab Glycoprofile	G0F-GlcNAc	G1F-GlcNAc	Man5	Man 7/9	G0F	G0	G1F	G2F	NA3
Hiatt *et al* [25]			0		39	5	37	8	
Palivizumab from *Nicotania benthamiana*[25]				17		76			
HPAEC-PAD (this work)	2.5	3.1	4.2		24.6		45.8	16.8	2.4

C

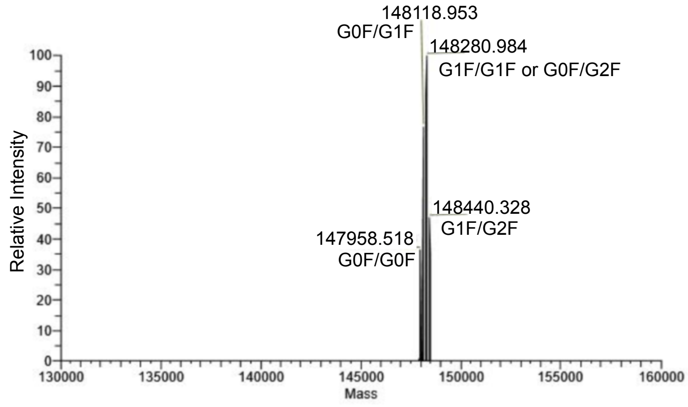

D

Commercial Palivizumab glycoform	Theoretical Mass (Da)	Observed Mass (Da)	Mass Error (ppm)
**G0F/G0F**	147,960.13	147,958.52	-8.87
**G0F/G1F**	148,122.27	148,118.95	-22.37
**G1F/G1F or G0F/G2F**	148,284.41	148,280.98	-23.09
**G1F/G2F**	148,446.55	148,440.33	-41.89

Supplemental Figure 1. *N*-glycan characterization of palivizumab. (**A**) HPAEC-PAD chromatogram of commercially-available palivizumab and (**B**) glycan analysis of palivizumab obtained commercially, compared to those reported in literature (25) (**C**) Intact mass spectrometry analysis of commercially available palivizumab shows the presence of various glycoforms on the same mAb (*i.e.* on each heavy chain of the mAb dimer). (**D**) Theoretical and observed intact mass values (Da) and mass error (ppm) of various glycoforms in commercial palivizumab.

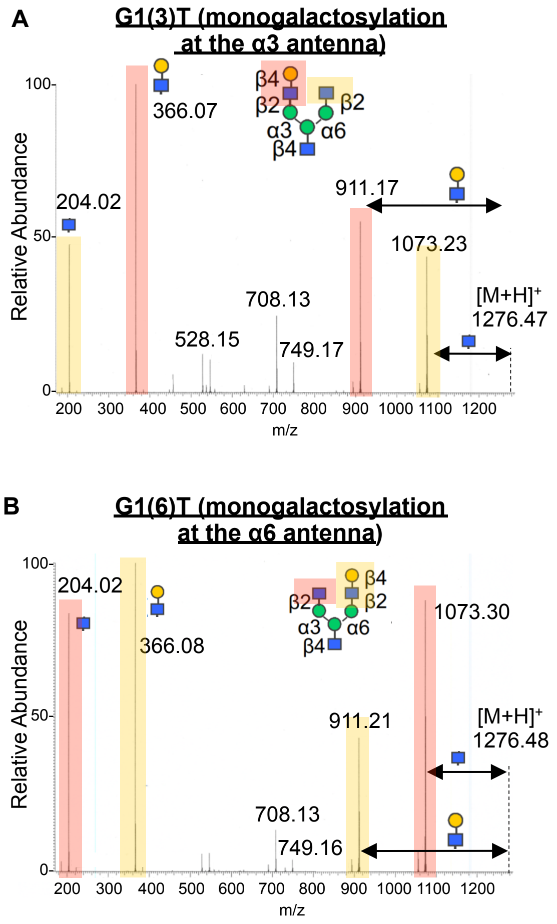

Supplementary Figure S2: Mass spectrometry characterization of branch-specific truncated monogalactosylated isomers (**A**) G1(3)T and (**B**) G1(6)T, obtained following purification by PGC-LC-HPLC. MS/MS spectra are shown at the doubly charged ions of m/z 638.7374 and 638.7375 for (A) and (B), respectively. Cleavage of the α3 antenna between the GlcNAc-β1-2-Man linkage produces a relatively higher abundance of the cleaved and its complementary glycan fragment compared to cleavage of the α6 antenna.

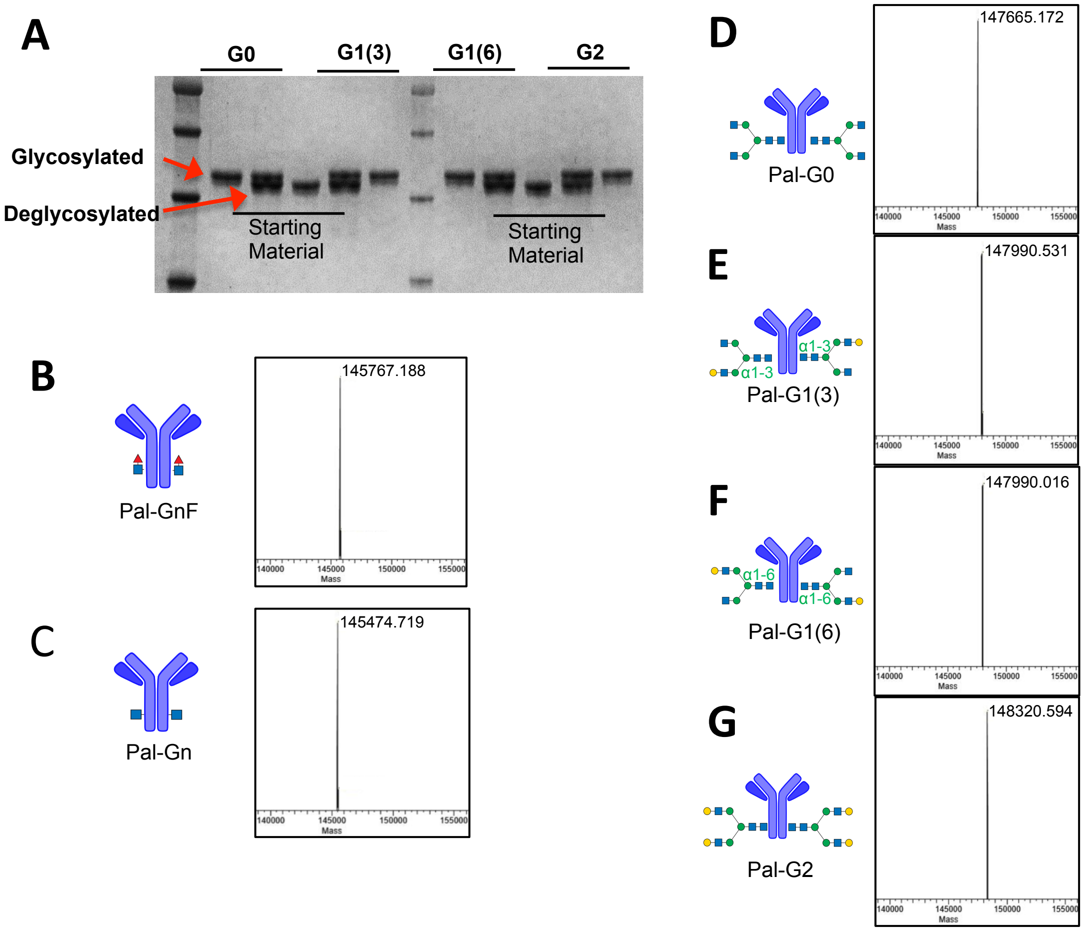

H

Palivizumab glycoform	Theoretical Mass (Da)	Observed Mass (Da)	Mass Error (ppm)
**Pal-GnF**	145,764.61	145,767.19	17.70
**Pal-Gn**	145,472.33	145,474.72	16.42
**Pal-G0**	147,664.33	147,665.17	5.70
**Pal-G1(3)**	147,988.61	147,990.53	12.98
**Pal-G1(6)**	147,988.61	147,990.02	9.50
**Pal-G2**	148,312.89	148,320.59	51.94

Supplemental Figure S3: Characterization of defucosylated palivizumab analogues with varying degrees of galactosylation. (**A**) SDS-PAGE analysis shows the shift in bands between starting material (deglycosylated) and completed reaction (glycosylated). (**B-G**) Deconvoluted intact mass spectrometry spectra shows the presence of palivizumab (**B**) following deglycosylation with EndoS, (**C**) Fucosidase GH29, then transglycosylation with EndoS D233Q and purified oxazoline glycans to form homogeneous palivizumabs comprising (**D**) agalactosylated G0, (**E**) monogalactosylation at the α3 antenna (G1(3)), (**F**) monogalactosylation at the α6 antenna (G1(6)), or(**G**) digalactosylated G2. (**H**) Theoretical and observed intact mass values (Da) and mass error (ppm) of various palivizumab glycoform intermediates and final products following enzymatic transglycosylation.

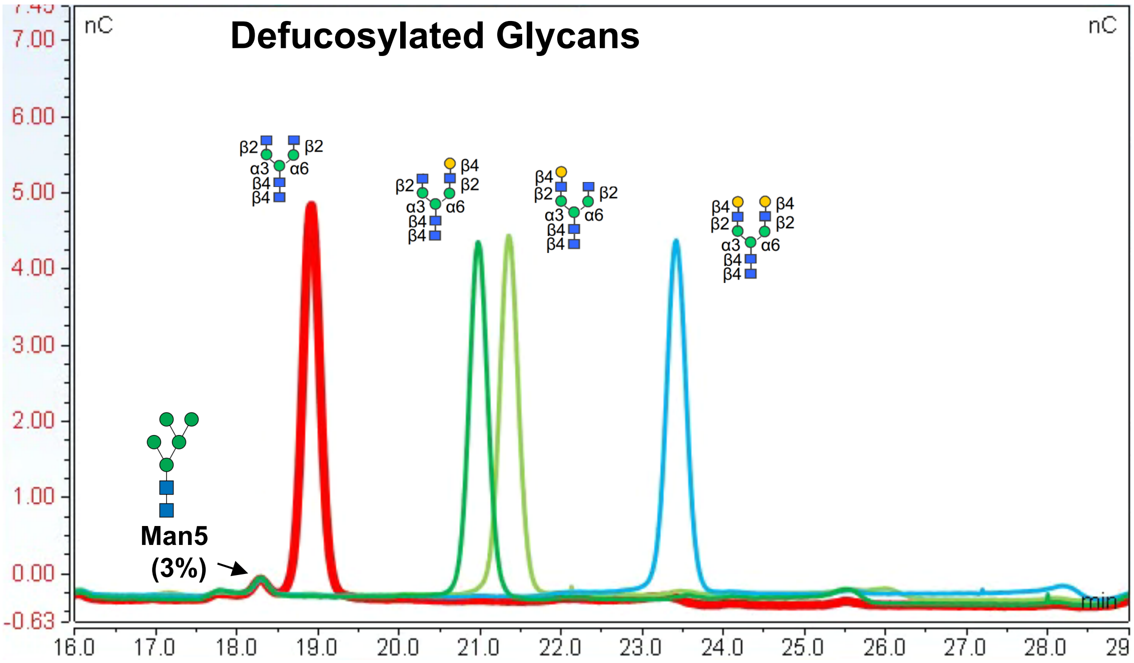

Supplementary Figure S4. HPAEC-PAD chromatogram of PNGase-F-released *N*-glycans from remodeled palivizumab. Un-remodelled Man5 glycans were 3 % for each analogue, indicating that enzymatic glycosylation is consistent for all remodelled analogues.

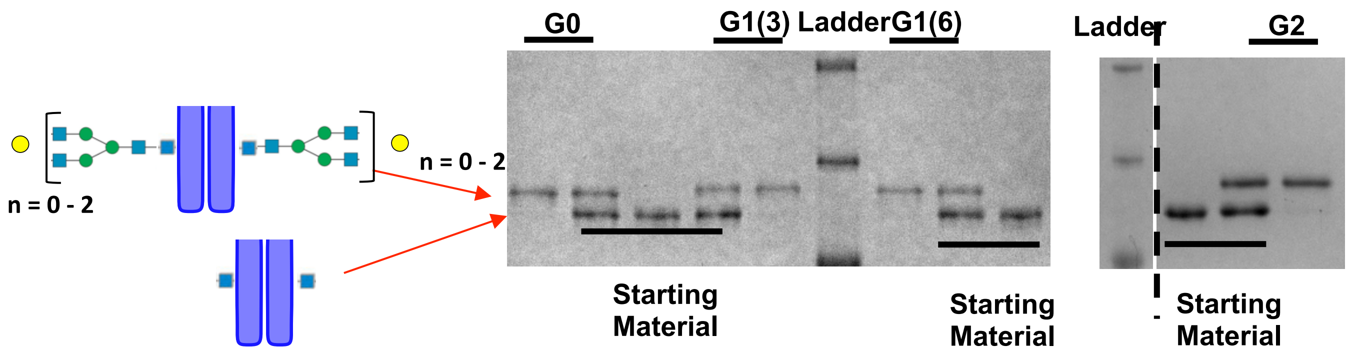

Supplemental Figure S5. Characterization of transglycosylated ^{15}N-NISTmAb-Fc with homogeneous glycoforms. Reducing SDS-PAGE gels show the shift in band migration of transglycosylated isotopically ^{15}N-NISTmAb-Fc (with G0, G1(3), G1(6), or G2) compared to starting material comprising only a single GlcNAc residue on each monomer. Lanes showing two bands contain both starting material and the transglycosylated Fc product to ensure these are two discreet bands.

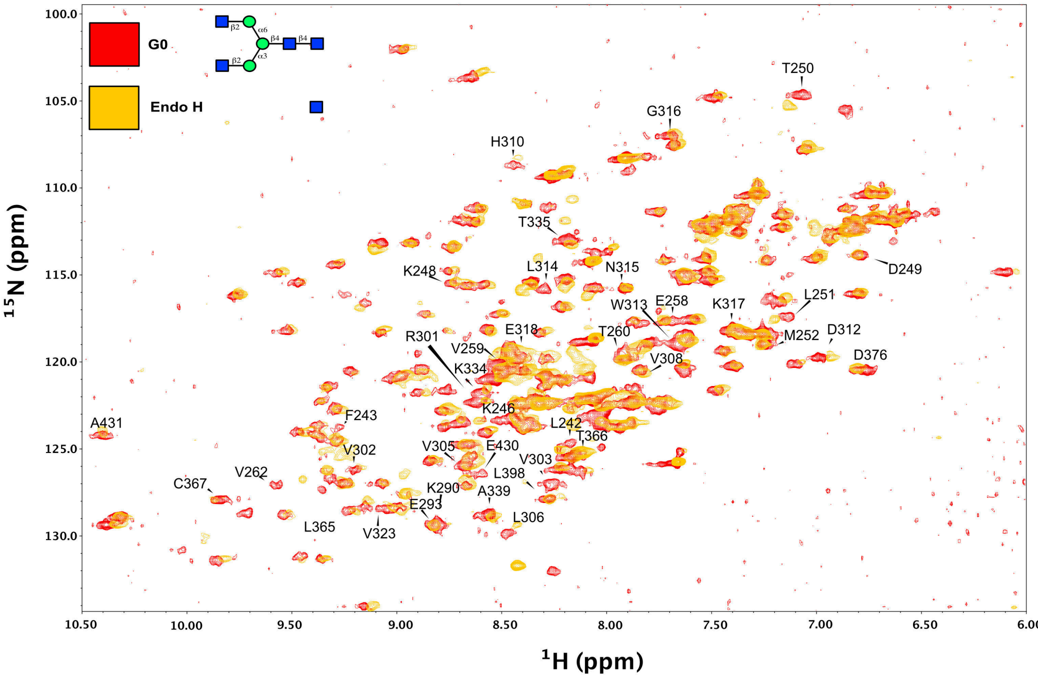

Supplemental Figure S6. Overlay of 2D ^{1}H-^{15}N NMR spectra of EndoH-treated ^{15}N-Gn-NISTmAb-Fc (orange) and remodelled ^{15}N-G0-NISTmAb-Fc (red).

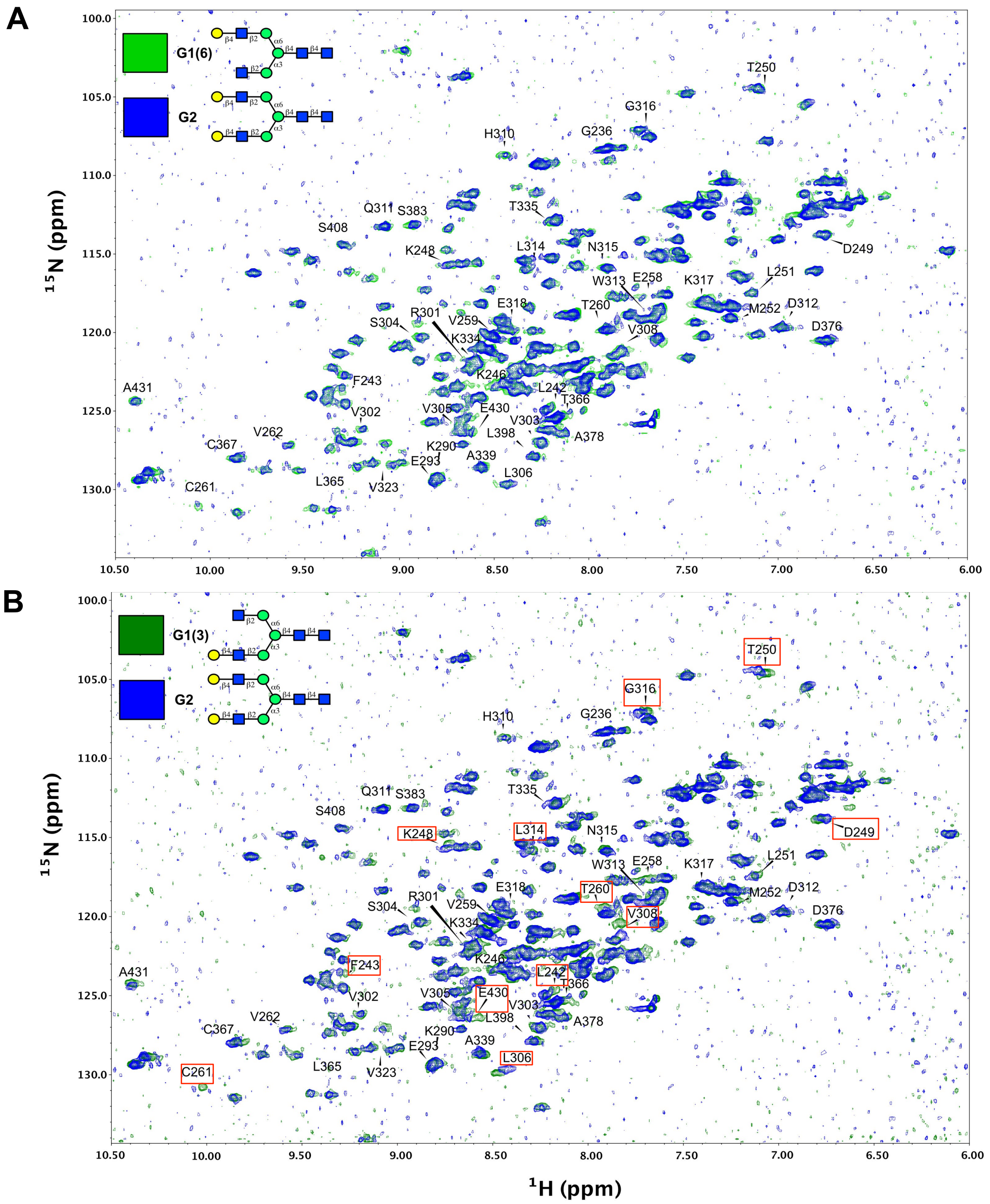

Supplemental Figure S7. Overlay of 2D ^{1}H-^{15}N NMR spectra of digalactosylated ^{15}N-G2-NISTmAb-Fc (blue) with (**A**) monogalactosylated remodelled ^{15}N-G1(6)-NISTmAb-Fc (bright green), and (**B**) ^{15}N-G1(3)-NISTmAb-Fc (dark green). Peptide backbone amino acid residues that show chemical shift perturbations (CSPs) are labelled and highlighted with a red box, while examples of other residues that show little to no CSPs are labelled as black text only without a red box. Compared to the digalactosylated Fc protein, more chemical shift perturbations are observed with the monogalactosylation at the α3 antenna (B) compared to the α6 antenna (A).

Supplemental Table S1 ^{1}H and ^{15}N chemical shifts (in ppm) and Combined Chemical Shift Difference (CCSD, in ppm) of amino acid residues that show Chemical Shift Perturbations (CSPs) between agalactosylated afucosylated Fc glycoform (G0) with glycoforms bearing a terminal galactose residue at the α6 antenna. Low CCSD values are observed amongst afucosylated glycoforms with a terminal galactose residue at the α6 antenna (i.e. G1(6) vs G2). D312 is used as a reference for a residue that showed low CCSD in all analogues.

Amino Acid Residue	Fc-Glycoform						CCSD (ppm)		
	G0		G1(6)		G2				
	Chemical Shift								
	δH (ppm)	δN (ppm)	δH (ppm)	δN (ppm)	δH (ppm)	δN (ppm)	G0 - G1(6)	G0 - G2	G1(6) - G2
**L242**	8.17	124.71	8.20	124.80	8.23	124.97	0.04	0.10	0.06
**F243**	9.27	123.78	9.31	123.54	9.31	123.51	0.08	0.09	0.01
**K248**	8.74	115.47	8.72	115.72	8.75	115.74	0.08	0.09	0.02
**D249**	6.79	113.87	6.75	113.75	6.75	113.76	0.06	0.05	0.00
**T250**	7.08	104.68	7.11	104.46	7.11	104.44	0.08	0.08	0.01
**T260**	7.92	119.50	7.91	119.86	7.91	119.86	0.11	0.11	0.00
**C261**	10.02	130.79	10.06	131.07	10.07	130.96	0.10	0.09	0.04
**S304**	8.88	120.41	8.88	120.32	8.89	120.33	0.03	0.03	0.01
**L306**	8.47	129.86	8.43	129.64	8.43	129.67	0.08	0.08	0.01
**V308**	7.84	121.49	7.83	121.17	7.83	121.22	0.10	0.08	0.01
**D312**	6.99	119.74	6.99	119.72	6.98	119.72	0.01	0.01	0.01
**L314**	8.29	115.89	8.34	115.98	8.34	116.02	0.05	0.06	0.01
**G316**	7.69	107.02	7.74	107.06	7.72	107.04	0.04	0.03	0.02
**A339**	8.56	128.81	8.56	128.59	8.57	128.57	0.07	0.07	0.01
**C367**	9.84	127.93	9.86	128.06	9.86	128.06	0.05	0.05	0.00
**A378**	8.12	126.24	8.13	126.41	8.12	126.38	0.05	0.05	0.01
